# Conditional Variational Autoencoder for Prediction and Feature Recovery Applied to Intrusion Detection in IoT

**DOI:** 10.3390/s17091967

**Published:** 2017-08-26

**Authors:** Manuel Lopez-Martin, Belen Carro, Antonio Sanchez-Esguevillas, Jaime Lloret

**Affiliations:** 1 Dpto. TSyCeIT, ETSIT, Universidad de Valladolid, Paseo de Belén 15, 47011 Valladolid, Spain; manuel.lopezm@uva.es (M.L.-M.); antoniojavier.sanchez@uva.es (A.S.-E.); 2 Instituto de Investigación para la Gestión Integrada de Zonas Costeras, Universitat Politècnica de València, Camino Vera s/n, 46022 Valencia, Spain; jlloret@dcom.upv.es

**Keywords:** intrusion detection, variational methods, conditional variational autoencoder, feature recovery, neural networks

## Abstract

The purpose of a Network Intrusion Detection System is to detect intrusive, malicious activities or policy violations in a host or host’s network. In current networks, such systems are becoming more important as the number and variety of attacks increase along with the volume and sensitiveness of the information exchanged. This is of particular interest to Internet of Things networks, where an intrusion detection system will be critical as its economic importance continues to grow, making it the focus of future intrusion attacks. In this work, we propose a new network intrusion detection method that is appropriate for an Internet of Things network. The proposed method is based on a conditional variational autoencoder with a specific architecture that integrates the intrusion labels inside the decoder layers. The proposed method is less complex than other unsupervised methods based on a variational autoencoder and it provides better classification results than other familiar classifiers. More important, the method can perform feature reconstruction, that is, it is able to recover missing features from incomplete training datasets. We demonstrate that the reconstruction accuracy is very high, even for categorical features with a high number of distinct values. This work is unique in the network intrusion detection field, presenting the first application of a conditional variational autoencoder and providing the first algorithm to perform feature recovery.

## 1. Introduction

A Network Intrusion Detection System (NIDS) is a system which detects intrusive, malicious activities or policy violations in a host or host’s network. The importance of NIDS is growing as the heterogeneity, volume and value of network data continue to increase. This is especially important for current Internet of Things (IoT) networks [1], which carry mission-critical data for business services.

Intrusion detection systems can be host-based or network-based. The first monitor and analyze the internals of a computer system while the second deal with attacks on the communication interfaces [2]. For this work, we will focus on network-based systems.

Intruders in a system can be internal or external. Internal intruders have access to the system but their privileges do not correspond to the access made, while the external intruders do not have access to the system. These intruders can perform a great variety of attacks: denial of service, probe, user to root attacks, etc. [2].

NIDS has been a field of active research for many years, being its final goal to have fast and accurate systems able to analyze network traffic and to predict potential threats. It is possible to classify NIDS by detection approach as signature-based detection approaches and anomaly-based detection methods. Signature-based detection methods use a database of previously identified bad patterns to identify and report an attack, while anomaly-based detection uses a model to classify (label) traffic as good or bad, based mainly on supervised or unsupervised machine learning methods. One characteristic of anomaly-based methods is the need to deal with unbalanced data. This happens because intrusions in a system are usually an exception, difficult to separate from the usually more abundant normal traffic. Working with unbalanced data is often a challenge for both the prediction algorithms and performance metrics used to evaluate systems. 

There are different ways to set up an intrusion detection model [3], adopting different approaches: probabilistic methods, clustering methods or deviation methods. In probabilistic methods, we characterize the probability distribution of normal data and define as an anomaly any data with a given probability lower than a threshold. In clustering methods, we cluster the data and categorize as an anomaly any data too far away from the desired normal data cluster. In deviation methods, we define a generative model able to reconstruct the normal data, in this setting we consider as an anomaly any data that is reconstructed with an error higher than a threshold. 

For this work, we present a new anomaly-based supervised machine learning method. We will use a deviation-based approach, but, instead of designating a threshold to define an intrusion, we will use a discriminative framework that will allow us to classify a particular traffic sample with the intrusion label that achieves less reconstruction error. We call the proposed method Intrusion Detection CVAE (ID-CVAE). The proposed method is based on a conditional variational autoencoder (CVAE) [4,5] where the intrusion labels are included inside the CVAE decoder layers. We use a generative model based on variational autoencoder (VAE) concepts, but relying on two inputs: the intrusion features and the intrusion class labels, instead of using the intrusion features as a single input, as it is done with a VAE. This change provides many advantages to our ID-CVAE when comparing it with a VAE, both in terms of flexibility and performance.

When using a VAE to build a classifier, it is necessary to create as many models as there are distinct label values, each model requiring a specific training step (one vs. rest). Each training step employs, as training data, only the specific samples associated with the label learned, one at a time. Instead, ID-CVAE needs to create a single model with a single training step, employing all training data irrespective of their associated labels. This is why a classifier based on ID-CVAE is a better option in terms of computation time and solution complexity. Furthermore, it provides better classification results than other familiar classifiers (random forest, support vector machines, logistic regression, multilayer perceptron), as we will show in Section 4.1.

ID-CVAE is essentially an unsupervised technique trained in a supervised manner, due to the use of class labels during training. More important than its classification results, the proposed model (ID-CVAE) is able to perform feature reconstruction (data recovery). ID-CVAE will learn the distribution of features values by relying on a mapping to its internal latent variables, from which a later feature recovery can be performed in the case of input samples with incomplete features. In particular, we will show that ID-CVAE is able to recover categorical features with accuracy over 99%. This ability to perform feature recovery can be an important asset in an IoT network. IoT networks may suffer from connection and sensing errors that may render some of the received data invalid [6]. This may be particularly important for categorical features that carry device`s state values. The work presented in this paper allows recovering those missing critical data, as long as we have available some related features, which may be less critical and easier to access (Section 4.2).

This work is unique in the NIDS field, presenting the first application of a conditional VAE and providing the first algorithm to perform feature recovery. The paper is organized as follows: Section 2 presents related works. Section 3 describes the work performed. Section 4 describes the results obtained and, finally, Section 5 provides conclusion and future work.

## 2. Related Works

As far as we know, there is no previous reported application of a CVAE to perform classification with intrusion detection data, although there are works related with VAE and CVAE in other areas.

An and Cho [7] presented a classifier solution using a VAE in the intrusion detection field, but it is a VAE (not CVAE) with a different architecture to the one presented here. They use the KDD 99 dataset. The authors of [4] apply a CVAE to a semi-supervised image classification problem. In [8] they used a recurrent neural network (RNN) with a CVAE to perform anomaly detection on one Apollo’s dataset. It is applied to generic multivariate time-series. The architecture is different to the one presented and the results are not related to NIDS. Similarly [9] employs an RNN with a VAE to perform anomaly detection on multivariate time-series coming from a robot. Data and results are not applicable to NIDS.

There are works that present results applying deep learning models to classification in the intrusion detection field. In [10] a neural network is used for detecting DoS attacks in a simulated IoT network, reporting an accuracy of 99.4%. The work in [11] presents a classifier which detects intrusions in an in-vehicle Controller Area Network (CAN), using a deep neural network pre-trained with a Deep Belief Network (DBN). The authors of [12] use a stacked autoencoder to detect multilabel attacks in an IEEE 802.11 network with an overall accuracy of 98.6%. They use a sequence of sparse auto-encoders but they do not use variational autoencoders. Ma et al. [13] implemented an intrusion classifier combining spectral clustering and deep neural networks in an ensemble algorithm. They used the NSL-KDD dataset in different configurations, reporting an overall accuracy of 72.64% for a similar NSL-KDD configuration to the one presented in this paper.

Using other machine learning techniques, there is also an important body of literature applying classification algorithms to the NSL-KDD dataset. It is important to mention that comparison of results in this field is extremely difficult due to: (1) the great variability of the different available datasets and algorithms applied; (2) the aggregation of classification labels in different sets (e.g., 23 labels can be grouped hierarchically into five or two subsets or categories); (3) diversity of reported performance metrics and (4) reporting results in unclear test datasets. This last point is important to mention, because for example, for the NSL-KDD dataset, 16.6% of samples in the test dataset correspond to labels not present in the training dataset. This is an important property of this dataset and creates an additional difficulty to the classifier. From this, it is clear how the performance of the classification may be different if the prediction is based on a subset of the training or test datasets, rather than the complete set of test data. 

The difficulties presented above are shown in detail in [14]. In [15], applying a multilayer perceptron (MLP) with three layers to the NSL-KDD dataset, they achieved an accuracy of 79.9% for test data, for a 5-labels intrusion scenario. For a 2-labels (normal vs. anomaly) scenario they provided an accuracy of 81.2% for test data. In [16] they provided, for a 2-labels scenario and using self-organizing maps (SOM), a recall of 75.49% on NSL-KDD test data. The authors of [17] reported employing AdaBoost with naive Bayes as weak learners, an F1 of 99.3% for a 23-labels scenario and an F1 of 98% for a 5-labels scenario; to obtain these figures they used 62,984 records for training (50% of NSL-KDD), where 53% are normal records and the remaining 47% are distributed among the different attack types; test results are based on 10-fold cross-validation over the training data, not on the test set. Bhuyan et al. [2] explained the reasons for creating the NSL-KDD dataset. They gave results for several algorithms. The best accuracy reported was 82.02% with naive Bayes tree using Weka. They use the full NSL_KDD dataset for training and testing, for the 2-labels scenario.

ID-CVAE’s ability to recover missing features is unique in the literature. There are other applications of generative models to NIDS, but none of them reports capabilities to perform feature recovery. In [18,19], the authors used a generative model—a Hidden Markov Model—to perform classification only. The work in [18] does not report classification metrics and [19] provides a precision of 93.2% using their own dataset. In [20] they resorted to a deep belief network applied to the NSL-KDD dataset to do intrusion detection. They reported a detection accuracy of 97.5% using just 40% of the training data, but it is unclear what test dataset is used. Xu et al. [21] employed continuous time Bayesian networks as detection algorithm, using the 1998 DARPA dataset. They achieved good results on the 2-labels scenario; the metric provided is a ROC curve. Finally, [22] presents a survey of works related to neural networks architectures applied to NIDS, including generative models; but no work on feature recovery is mentioned.

Using a different approach, [6] proposes a method to recover missing (incomplete) data from sensors in IoT networks using data obtained from related sensors. The method used is based on a probabilistic matrix factorization and it is more applicable to the recovery of continuous features. Related to NIDS for IoT, specifically wireless sensor networks, Khan et al. [23] presents a good review of the problem, and [24,25] show details of some of the techniques applied.

## 3. Work Description 

In the following sections, we present the dataset used for this work and a description of the variational Bayesian method that we have employed.

### 3.1. Selected Dataset

We have used the NSL-KDD dataset as a representative dataset for intrusion detection. The NSL-KDD [14] dataset is a derivation of the original KDD 99 dataset. It solves the problem of redundant samples in KDD 99, being more useful and realistic. NSL-KDD provides a sufficiently large number of samples. The distribution of samples among intrusion classes (labels) is quite unbalanced and provides enough variability between training and test data to challenge any method that tries to reproduce the structure of the data. 

The NSL-KDD dataset has 125,973 training samples and 22,544 test samples, with 41 features, being 38 continuous and three categorical (discrete valued) [15]. Six continuous variables were discarded since they contained mostly zeros. We have performed an additional data transformation: scaling all continuous features to the range [0–1] and one-hot encoding all categorical features. This provides a final dataset with 116 features: 32 continuous and 84 with binary values ({0, 1}) associated to the three one-hot encoded categorical features. It is interesting to note that the three categorical features: *protocol*, *flag*, and *service* have respectively three, 11 and 70 distinct values.

The training dataset contains 23 possible labels (normal plus 22 labels associated with different types of intrusion); meanwhile, the test dataset has 38 labels. That means that the test data has anomalies not present at training time. The 23 training and 38 testing labels have 21 labels in common; two labels only appear in training set and 17 labels are unique to the testing data. Up to 16.6% of the samples in the test dataset correspond to labels unique to the test dataset, and which were not present at training time. This difference in label distribution introduces an additional challenge to the classifiers. 

As presented in [14], the training/testing labels are associated to one of five possible categories: NORMAL, PROBE, R2L, U2R and DoS. All the above categories correspond to an intrusion except the NORMAL category, which implies that no intrusion is present. We have considered these five categories as the final labels driving our results. These labels are still useful to fine-grain characterize the intrusions, and are still quite unbalanced (an important characteristic of intrusion data) yet contain a number of samples, in each category, big enough to provide more meaningful results.

We use the full training dataset of 125,973 samples and the full test dataset of 22,544 samples for any result we provide concerning the training and test NSL-KDD datasets. It is also important to mention that we do not use a previously constructed (customized) training or test datasets, nor a subset of them, what may provide better-alleged results but be less objective and also miss the point to have a common reference to compare results.

### 3.2. Methodology

**Figure 1:**
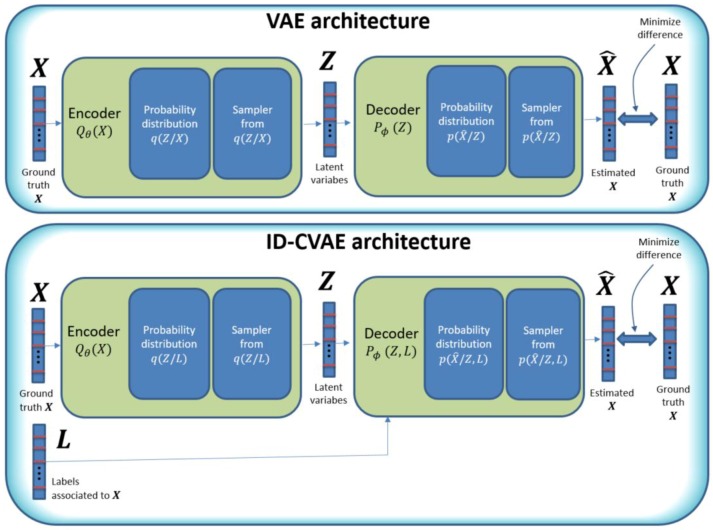
Comparison of ID-CVAE with a typical VAE architecture.

In Figure 1 we compare ID-CVAE and VAE architectures. In the VAE architecture [26], we try to learn the probability distribution of data: ***X***, using two blocks: an encoder and a decoder block. The encoder implements a mapping from ***X*** to a set of parameters that completely define an associated set of intermediate probability distributions: q(Z/X). These intermediate distributions are sampled, and the generated samples constitute a set of latent variables: Z, which forms the input to the next block: the decoder. The decoder block will operate in a similar way to the encoder, mapping from the latent variables to a new set of parameters defining a new set of associated probability distributions: $p\left(\hat{X}/Z\right)$, from which we take samples again. These final samples will be the output of our network: $\hat{X}$. 

The final objective is to approximate as much as possible the input and output of the network: X and $\hat{X}$. But, in order to attain that objective, we have to map the internal structure of the data to the probability distributions: q(Z/X) and $p\left(\hat{X}/Z\right)$.

The probability distributions $p\left(\hat{X}/Z\right)$ and q(Z/X) are conditional probability distributions, as they model the probability of $\hat{X}$ and Z but depend on their specific inputs: Z and X, respectively.

In a VAE, the way we learn the probability distributions: q(Z/X) and $p\left(\hat{X}/Z\right)$, is by using a variational approach [26], which translates the learning process to a minimization process, that can be easily formulated in terms of stochastic gradient descent (SGD) in a neural network. 

In Figure 1, the model parameters: θ and ϕ, are used as a brief way to represent the architecture and weights of the neural network used. These parameters are tuned as part of the VAE training process and are considered constant later on.

In the variational approach, we try to maximize the probability of obtaining the desired data as output, by maximizing a quantity known as the Evidence Lower Bound (ELBO) [22]. The ELBO is formed by two parts: (1) a measure of the distance between the probability distribution q(Z/X) and some reference probability distribution of the same nature (actually a prior distribution for Z), where the distance usually used is the Kullback-Leibler (KL) divergence; and (2) the log likelihood of p(X) under the probability distribution $p\left(\hat{X}/Z\right)$, that is the probability to obtain the desired data (X) with the final probability distribution that produces $\hat{X}$.

All learned distributions are parameterized probability distributions, meaning that they are completely defined by a set of parameters (e.g., the mean and variance of a normal distribution). This is very important in the operation of the model, as we rely on these parameters, obtained as network nodes values, to model the associated probability distributions: $p\left(\hat{X}/Z\right)$ and q(Z/X).

Based on the VAE model, our proposed method: ID-CVAE, has similarities to a VAE but instead of using exclusively the same data for the input and output of the network, we use additionally the labels of the samples as an extra input to the decoder block (Figure 1, lower diagram). That is, in our case, using the NSL-KDD dataset, which provides samples with 116 features and a class label with five possible values associated with each sample, we will have a vector of features (of length 116) as both input and output, and its associated label (one-hot encoded in a vector of length 5) as an extra input. 

To have the labels as an extra input, leads to the decoder probability distributions being conditioned on the latent variable and the labels (instead of exclusively on the latent variable: Z), while the encoder block does not change (Figure 1, lower diagram). This apparently small change, of adopting the labels as extra input, turns out to be an important difference, as it allows one to:

- Add extra information into the decoder block which is important to create the required binding between the vector of features and labels.
- Perform classification with a single training step, with all training data.
- Perform feature reconstruction. An ID-CVAE will learn the distribution of features values using a mapping to the latent distributions, from which a later feature recovery can be performed, in the case of incomplete input samples (missing features).

**Figure 2:**
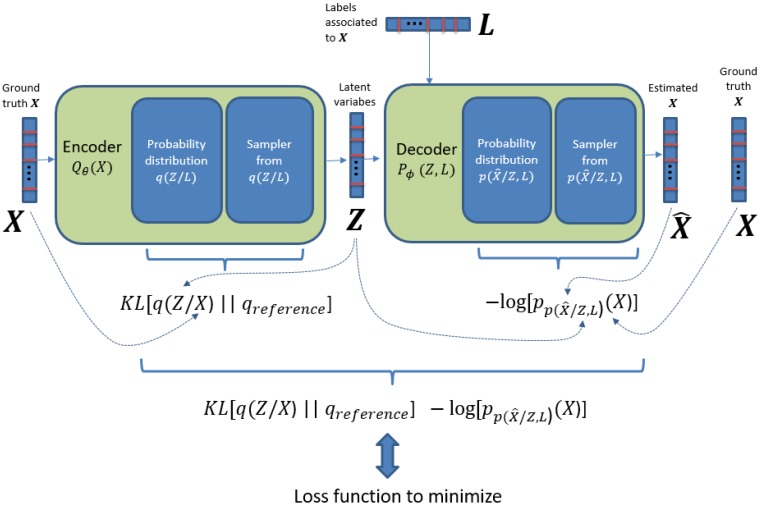
Details on the loss function elements for the ID-CVAE model.

In Figure 2 we present the elements of the loss function to be minimized by SGD for the ID-CVAE model. We can see that, as mentioned before, the loss function is made up of two parts: a KL divergence and a log likelihood part. The second part takes into account how probable is to generate X by using the distribution $p\left(\hat{X}/Z,L\right)$, that is, it is a distance between X and $\hat{X}$. The KL divergence part can be understood as a distance between the distribution q(Z/X) and a prior distribution for Z. By minimizing this distance, we are really avoiding that q(Z/X) departs too much from its prior, acting finally as a regularization term. The nice feature about this regularization term is that it is automatically adjusted, and it is not necessary to perform cross-validation to adjust a hyper-parameter associated to the regularization, as it is needed in other models (e.g., parameter λ in ridge regression).

### 3.3. Model Details

**Figure 3:**
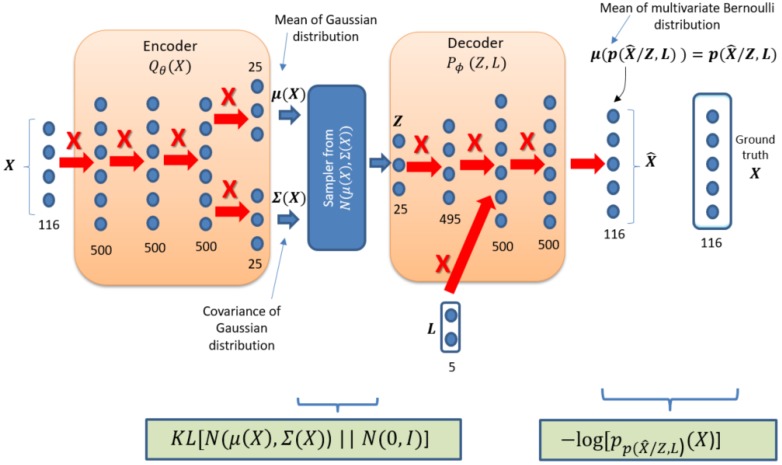
ID-CVAE model details.

The details of the ID-CVAE model are presented in Figure 3. We employ a multivariate Gaussian as the distribution for q(Z/X), with a mean μ(X) and a diagonal covariance matrix: $\Sigma \left(X\right)\to \;\sigma_{i}^{2}\left(X\right)$, with different values along the diagonal. We have a standard normal N(0,I) as the prior distribution for Z.

For the distribution $p\left(\hat{X}/Z,L\right)$ we use a multivariate Bernoulli distribution. The Bernoulli distribution has the interesting property of not requiring a final sampling, as the output parameter that characterizes the distribution is the mean that is the same as the probability of success. This probability can be interpreted as a [0–1] scaled value for the ground truth X, which has been already scaled to [0–1]. Then, in this case, the output of the last layer is taken as our final output $\hat{X}$.

The selection of distributions for q(Z/X) and $p\left(\hat{X}/Z,L\right)$ is aligned with the ones chosen in [26], they are simple and provide good results. The boxes at the lower part of Figure 3 show the specific choice of the loss function. This is a particular selection for the generic loss function presented in Figure 2.

An important decision is how to incorporate the label vector in the decoder. In our case, to get the label vector inside the decoder we just concatenate it with the values of the second layer of the decoder block (Figure 3). The position for inserting the L labels has been determined by empirical results (see Section 4.1) after considering other alternatives positions.

In Figure 3, a solid arrow with a nearby X designates a fully connected layer. The numbers behind each layer designate the number of nodes of the layer. The activation function of all layers is ReLU except for the activation function of last encoder layer that is Linear and the activation function of last decoder layer which is Sigmoid. The training has been performed without dropout.

## 4. Results

This section presents the results obtained by applying ID-CVAE and some other machine learning algorithms to the NSL-KDD dataset. A detailed evaluation of results is provided.

In order to appreciate the prediction performance of the different options, and considering the highly unbalanced distribution of labels, we provide the following performance metrics: accuracy, precision, recall, F1, false positive rate (FPR) and negative predictive value (NPV). We base our definition of these performance metrics on the usually accepted ones [2].

Considering all metrics, F1 can be considered the most important metric in this scenario. F1 is the harmonic mean of precision and recall and provides a better indication of prediction performance for unbalanced datasets. F1 gets its best value at 1 and worst at 0.

When doing either classification or feature reconstruction we will face a multi-class classification problem. There are two possible ways to give results in this case: aggregated and One-vs.-Rest results. For One-vs.-Rest, we focus in a particular class (label) and consider the other classes as a single alternative class, simplifying the problem to a binary classification task for each particular class (one by one). In the case of aggregated results, we try to give a summary result for all classes. There are different alternatives to perform the aggregation (micro, macro, samples, weighted), varying in the way the averaging process is done [27]. Considering the results presented in this paper, we have used the weighted average provided by scikit-learn [27], to calculate the aggregated F1, precision and recall scores.

### 4.1. Classification

**Figure 4:**
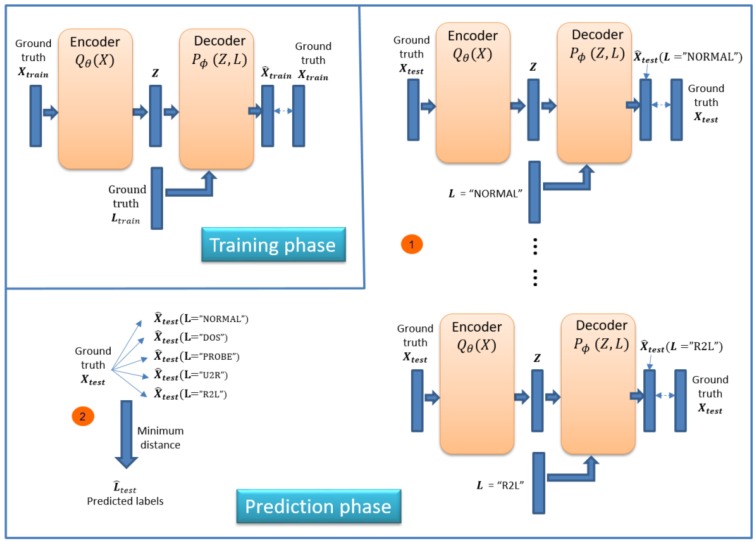
Classification framework.

We can use ID-CVAE as a classifier. Figure 4 shows the process necessary to perform classification. The process consists of two phases (in order): training and prediction phase. 

In the training phase, we train the model using a training dataset together with its associated labels. We train the model as presented in Section 3, trying to minimize the difference between the recovered and ground truth training dataset: ${\hat{X}}_{\mathrm{train}}$ vs. $X_{\mathrm{train}}$. 

In the prediction phase the objective is to retrieve the predicted labels for a new test dataset: $X_{\mathrm{test}}$. The prediction phase is made up of two steps (Figure 4). In the first step we apply the previously trained model to perform a forward pass to obtain a recovered test dataset $\left({\hat{X}}_{\mathrm{test}}\right)$. For this step, we use two inputs: the original test dataset plus a label vector with a single value. That is, we use as label input (L vector) a single label value (e.g., NORMAL, DOS, R2L) for all samples in the test dataset. We need to run this step as many times as there are distinct values in the label, each time changing the value of the L vector. This allows having a set of recovered test datasets, each one using a different label value.

In step two of the prediction phase, we calculate the distance between the ground truth test dataset and each of the recovered ones, choosing for each sample the label associated with the minimum distance. Several distances can be used and we have selected the Euclidean distance. 

The intuition behind this process is that the network learns how to recover the original features better when using the correct label as input. Therefore, we choose the label that generates the recovered features closer to the original ones.

For ID-CVAE, the classification process requires a single training stage followed by as many test stages as distinct values we try to predict. The training stage is the one demanding more time and resources, while the test stage is very light and fast. On the contrary, if we use a VAE to perform classification, we will require as many training and test stages as there are distinct label values. 

**Figure 5:**
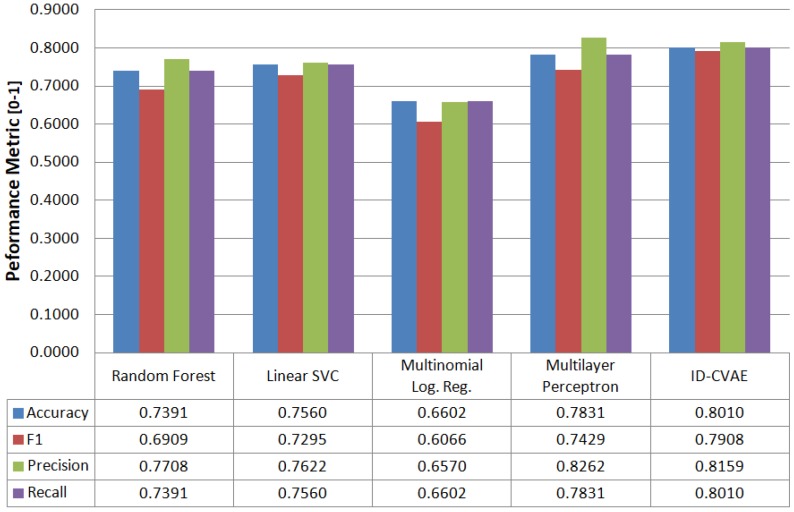
Classification performance metrics (aggregated) vs. different classifiers.

When applying the above-described process to the NSL-KDD test dataset we obtain the classification results presented in Figure 5. In Figure 5 we compare performance metrics for ID-CVAE with results obtained when applying conventional supervised algorithms: random forest, linear support vector machine (SVM), multinomial logistic regression and an MLP with two layers (200, 50). The results provided in Figure 5 are aggregated results. It can be seen that ID-CVAE presents the best overall results. In particular, ID-CVAE obtains an F1 score of 0.79 and an accuracy and recall of 0.80 which are the highest among the algorithms. The good results of ID-CVAE, compared with the alternative classifiers, indicate that ID-CVAE can better deals with the unbalanced and highly noisy data used in an NIDS. This behavior could be explained by the two-step process used to perform the classification, where the first step performs a stochastic data modeling and the second applies a discriminative approach to choose the best classification label. From the results, it seems that a combination of generative and discriminative methods is more appropriate for this kind of data.

**Figure 6:**
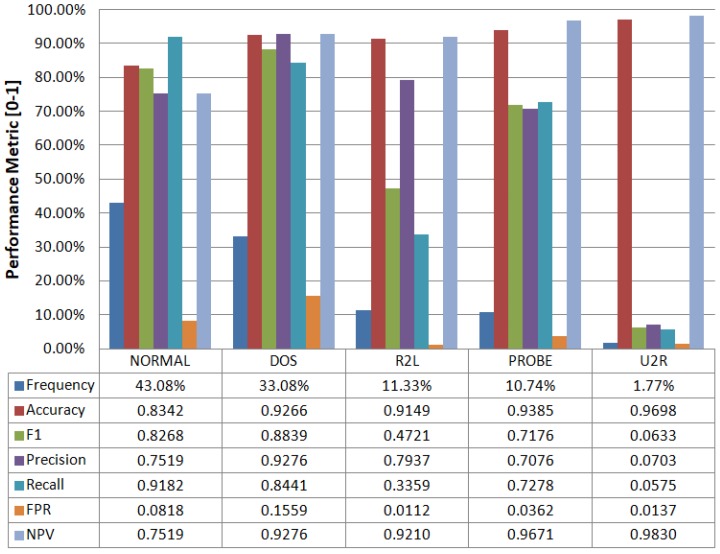
Classification performance metrics (One vs. Rest) vs. intrusion label.

Figure 6 shows one vs. rest detailed performance metrics for 5-labels classification using the ID-CAVE algorithm. We can observe how the frequency distribution for the labels is highly unbalanced (row “Frequency” in Figure 6). We get an F1 score greater than 0.8 for the most frequent labels. The behavior of lower frequency labels is quite noisy due to the nature of the training and test datasets. The accuracy obtained is always greater than 0.83 regardless of the label.

Table 1 presents the confusion matrix for the classification of five labels. The confusion matrix provides a sample count according to actual (ground-truth) and predicted labels. The table also provides totals and percentages along rows and columns. From Table 1, the number of correctly classified and misclassified samples can be easily obtained. In this table, we can see that the classification of the label R2L presents more difficulties due to the number of misclassifications. The U2R label has the worst results, which is expected given the very low frequency of this label (1.77%).

In Table 2 we present the empirical results to determine the position for inserting the labels in the decoder. The insertion in the second layer provides the best classification results, we can also observe that the position of insertion is an important element to consider when defining the architecture of the model.

All results presented in this Section (label frequency, confusion matrix, and performance metrics) are calculated using the full NSL-KDD test dataset.

### 4.2. Feature Reconstruction

**Figure 7:**
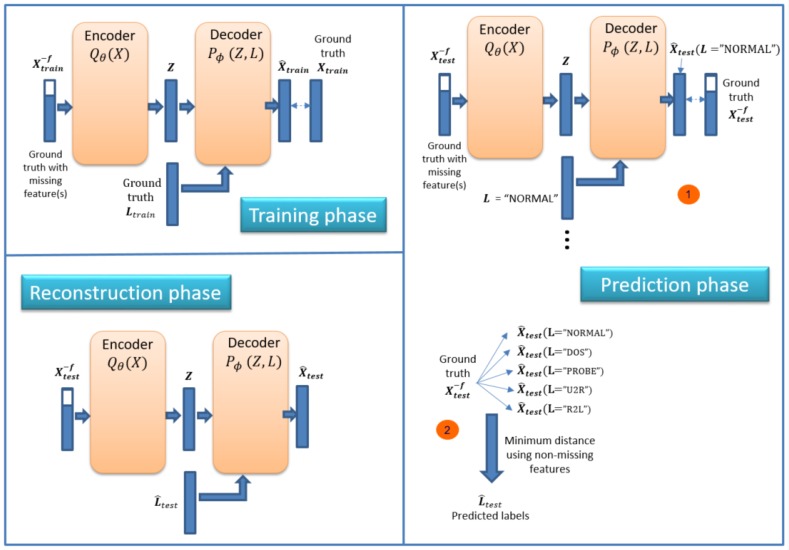
Feature reconstruction framework.

ID-CVAE can perform feature reconstruction. Figure 7 shows the process that consists of three phases (in order): training, prediction and reconstruction phase.

The objective here will be to reconstruct missing features in an intrusion detection test dataset, i.e., one with unknown labels. To do that, we start by training an ID-CVAE with the dataset with missing features (shown in Figure 7 as $X^{-f}$), but using the complete dataset as reference (Figure 7, training phase).

Then we need to perform a prediction phase (Figure 7). The input to this phase will be again the dataset with missing features. We do the prediction in a similar way as presented in Section 4.1, to perform classification; the only difference is that we will have a recovered test dataset with more features than the original one (used as reference). That creates a possible problem to perform the distance calculation between them. In order to solve this problem, we use only the non-missing features in both datasets to perform the distance calculation. 

Once the predicted labels are obtained, we use these labels together with the original test dataset (with missing features) as inputs to our model, having as output the recovered test dataset (with all features), as can be seen in Figure 7 (reconstruction phase).

It is important to note that we only need the training dataset for the training phase. This training dataset contains the full set of features, from which we extract a reduced set $X_{\mathrm{train}}^{-f}$ (a subset of the features from the training data) that will be used to train the model. After the training phase, we will only have access to a test dataset with missing features $X_{\mathrm{test}}^{-f}$ (the same missing features provided at the training phase). The ground truth $X_{\mathrm{test}}$ (with all features) is always unknown, and we try to approximate it in two phases: first, we use an initial reconstruction of the test dataset to predict the most probable associated labels ${\hat{L}}_{\mathrm{test}}$, and, second, using these labels we perform a final reconstruction ${\hat{X}}_{\mathrm{test}}$, that tries to recover the unknown ground truth $X_{\mathrm{test}}$.

**Figure 8:**
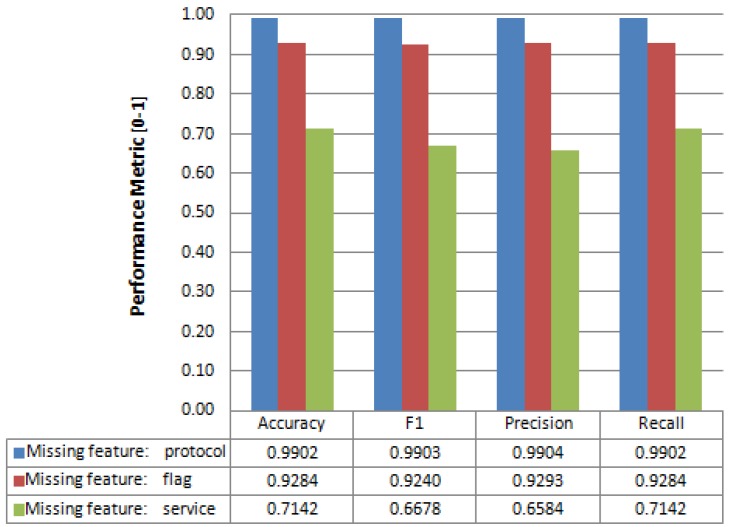
Performance metrics (aggregated) for predicting missing features of NSL-KDD test dataset.

When we apply the feature reconstruction process to the NSL-KDD Test dataset, we obtain the results given in Figure 8. This figure provides performance metrics when recovering some missing features; in particular: *protocol*, *flag* and *service* features; all of them categorical. As expected, the achievable prediction accuracy is related to the number of values of the categorical feature. The features *protocol* and *flag* with three and 11 values, respectively, have an accuracy of 99% and 92%, while the feature *service* with 70 values has an accuracy of 71%, which is not a bad result considering the large number of values of this feature. It would be necessary to obtain more training data to achieve a better result in the recovery of features with many values, due to the greater uncertainty associated with a label with more values. The remaining metrics (F1, precision, and recall) follow a similar pattern (Figure 8).

**Figure 9:**
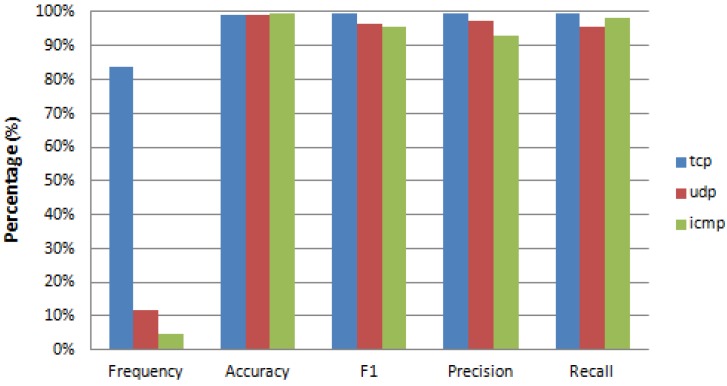
Performance metrics (One vs. Rest) for reconstruction of all features values when feature: ‘protocol’ is missing.

**Figure 10:**
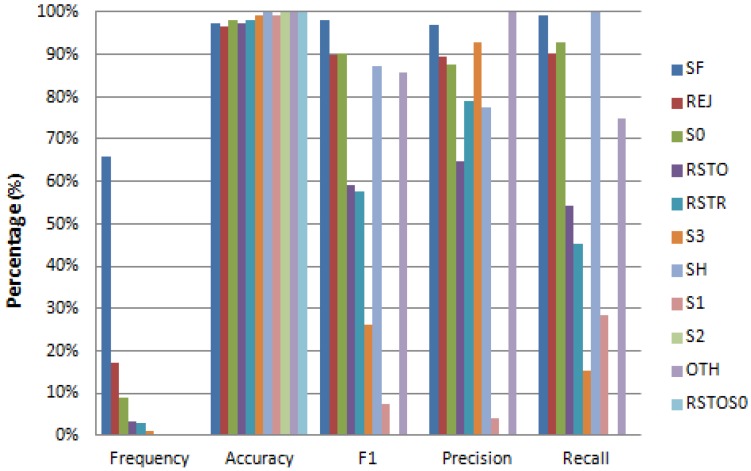
Performance metrics (One vs. Rest) for reconstruction of all features values when feature: ‘flag’ is missing.

**Figure 11:**
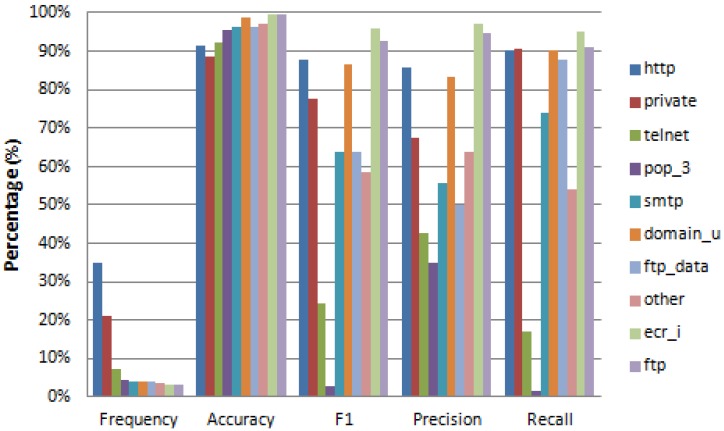
Performance metrics (One vs. Rest) for reconstruction of all features values when feature: ‘*service*’ is missing.

Figure 9, Figure 10 and Figure 11 provide detailed results on reconstruction performance for each of the three recovered features (*protocol*, *flag*, and *service*). Each figure provides the name and frequency distribution for feature’s values, together with one vs. rest reconstruction metrics. The reconstruction metrics are given for each value of the reconstructed feature (one vs. rest). We can see that in all cases the frequency distribution of values is remarkably unbalanced (column “Frequency” in Figure 9, Figure 10 and Figure 11). The unbalanced distribution creates additional problems to the recovery task. There are cases where this unbalanced scenario is so strong that the algorithm cannot recover certain values of the reconstructed feature. This is the case in Figure 10, where several rare values of the *flag* feature (S1, S2, and RSTOS0) have an F1 equal or close to zero, implying that we cannot predict any positive occurrence of these values. This happens for values with extremely low frequency (less than 0.09%). 

Figure 9 and Figure 10 present results for the recovery metrics for all values of the *protocol* and *flag* features (with three and 11 values, respectively) and Figure 11 presents results for the 10 most frequent values of the *service* feature, which has 70 values in total.

In Figure 9, when recovering the *protocol* feature we can achieve an F1 score of not less than 0.96 for any value of the feature, regardless of its frequency.

While recovering the *flag* feature (Figure 10), we obtain an accuracy always greater than 0.97 and an F1 score greater than 0.9 for the most frequent values.

Similarly, when recovering the *service* feature, we get an accuracy greater than 0.89 for the 10 most frequent values of this feature and a noisy F1 score with a higher value of 0.96.

In Table 3, we present the confusion matrix for the recovery of the three values of the feature: ‘protocol’. This is information similar to that given for the classification case (Section 4.1). Table 4 also shows detailed performance metrics such as those provided for the classification case.

We only present detailed data (as in Table 3 and Table 4) for the case of recovery of the feature: ‘protocol’. Similar data could be presented for the other two discrete features, but their large number of values would provide too much information to be useful for analysis.

The description of the data presented in Table 3 and Table 4 is similar to the data presented in Table 1 and Figure 6.

So far, we have only covered the reconstruction of discrete features. However, we have also done the experiment to recover all continuous features using only the three discrete features to perform the recovery. We obtained a Root Mean Square Error (RMSE) of 0.1770 when retrieving the 32 continuous features from the discrete features. All performance metrics are calculated using the full NSL-KDD test dataset.

### 4.3. Model Training

These are lessons learned about training the models: The inclusion of drop-out as regularization gives worse results. Having more than two or three layers for the encoder or decoder does not improve the results, making the training more difficult. It is important to provide a fair number of epochs for training the models, usually 50 or higher.

We have used Tensorflow to implement all the ID-CVAE models, and the python package scikit-learn [27] to implement the different classifiers. All computations have been performed on a commercial PC (i7-4720-HQ, 16 GB RAM).

## 5. Conclusions and Future Work

This work is unique in presenting the first application of a conditional VAE (CVAE) to perform classification on intrusion detection data. More important, the model is also able to perform feature reconstruction, for which there is no previous published work. Both capabilities can be used in current NIDS, which are part of network monitoring systems, and particularly in IoT networks [1].

We have demonstrated that the model performs extremely well for both tasks, being able for example to provide better classification results on the NSL-KDD Test dataset than well-known algorithms: random forest, linear SVM, multinomial logistic regression and multi-layer perceptron.

The model is also less complex than other classifier implementations based on a pure VAE. The model operates creating a single model in a single training step, using all training data irrespective of their associated labels. While a classifier based on a VAE needs to create as many models as there are distinct label values, each model requiring a specific training step (one vs. rest). Training steps are highly demanding in computational time and resources. Therefore, reducing its number from n (number of labels) to 1 is an important improvement.

When doing feature reconstruction, the model is able to recover missing categorical features with three, 11 and 70 values, with an accuracy of 99%, 92%, and 71%, respectively. The reconstructed features are generated from a latent multivariate probability distribution whose parameters are learned as part of the training process. This inferred latent probability distribution serves as a proxy for obtaining the real probability distribution of the features. This inference process provides a solid foundation for synthesizing features as similar as possible to the originals. Moreover, by adding the sample labels, as an additional input to the decoder, we improve the overall performance of the model making its training easier and more flexible.

Extensive performance metrics are provided for multilabel classification and feature reconstruction problems. In particular, we provide aggregated and one vs. rest metrics for the predicted/reconstructed labels, including accuracy, F1 score, precision and recall metrics.

Finally, we have presented a detailed description of the model architecture and the operational steps needed to perform classification and feature reconstruction.

Considering future work, after corroborating the good performance of the conditional VAE model, we plan to investigate alternative variants as ladder VAE [28] and structured VAE [29], to explore their ability to learn the probability distribution of NIDS features.

## Figures and Tables

**Table 1:** Classification confusion matrix.

		Prediction						
		DoS	Normal	Probe	R2L	U2R	Total	Percentage (%)
	**DoS**	6295	916	61	162	24	7458	33.08%
	**Normal**	119	8917	610	36	29	9711	43.08%
	**Probe**	368	252	1762	18	21	2421	10.74%
**Ground Truth**	**R2L**	4	1430	32	858	230	2554	11.33%
	**U2R**	0	345	25	7	23	400	1.77%
	**Total**	6786	11860	2490	1081	327	22544	100.00%
	**Percentage** **(%)**	30.10%	52.61%	11.05%	4.80%	1.45%	100.00%	

**Table 2:** Impact of layer used in the decoder to insert the labels.

Model	Accuracy	F1	Precision	Recall
**Labels inserted in first layer of decoder**	0.7791	0.7625	0.7888	0.7791
**Labels inserted in second layer of decoder (ID-CVAE)**	0.8010	0.7908	0.8159	0.8010
**Labels inserted in third layer of decoder**	0.7547	0.7389	0.7584	0.7547

**Table 3:** Confusion matrix for reconstruction of all features values when feature: ‘*protocol’* is missing.

		Prediction				
		icmp	tcp	udp	Total	Percentage (%)
**Ground Truth**	**icmp**	1022	19	2	1043	4.63%
	**tcp**	13	18791	76	18880	83.75%
	**udp**	7	79	2535	2621	11.63%
	**Total**	1042	18889	2613	22544	100.00%
	**Percentage (%)**	4.62%	83.79%	11.59%	100.00%	

**Table 4:** Detailed performance metrics for reconstruction of all features values when feature: ‘*protocol’* is missing.

Label Value	Frequency	Accuracy	F1	Precision	Recall	FPR	NPV
tcp	83.75%	0.9917	0.9950	0.9948	0.9953	0.0267	0.9757
udp	11.63%	0.9927	0.9687	0.9701	0.9672	0.0039	0.9957
icmp	4.63%	0.9982	0.9803	0.9808	0.9799	0.0009	0.9990
